# Penetration State Recognition during Laser Welding Process Control Based on Two-Stage Temporal Convolutional Networks

**DOI:** 10.3390/ma17184441

**Published:** 2024-09-10

**Authors:** Zhihui Liu, Shuai Ji, Chunhui Ma, Chengrui Zhang, Hongjuan Yu, Yisheng Yin

**Affiliations:** 1 Joint SDU-NTU Centre for Artificial Intelligence Research, School of Software, Shandong University, Jinan 250101, China; 202220802@mail.sdu.edu.cn; 2 Key Laboratory of High Efficiency and Clean Mechanical Manufacture, School of Mechanical Engineering, Shandong University, Jinan 250061, Chinayinyisheng@mail.sdu.edu.cn (Y.Y.); 3 School of Mechanical Engineering, Shandong University, Jinan 250061, China; 4 China National Heavy Duty Truck Group, Jinan 250013, China

**Keywords:** weld penetration monitoring, laser welding, image processing, weld process control

## Abstract

Vision-based laser penetration control has become an important research area in the field of welding quality control. Due to the complexity and large number of parameters in the monitoring model, control of the welding process based on deep learning and the reliance on long-term information for penetration identification are challenges. In this study, a penetration recognition method based on a two-stage temporal convolutional network is proposed to realize the online process control of laser welding. In this paper, a coaxial vision welding monitoring system is built. A lightweight segmentation model, based on channel pruning, is proposed to extract the key features of the molten pool and the keyhole from the clear molten pool keyhole image. Using these molten pool and keyhole features, a temporal convolutional network based on attention mechanism is established. The recognition method can effectively predict the laser welding penetration state, which depends on long-term information. In addition, the penetration identification experiment and closed-loop control experiment of unequal thickness plates are designed. The proposed method in this study has an accuracy of 98.96% and an average inference speed of 20.4 ms. The experimental results demonstrate that the proposed method exhibits significant performance in recognizing the penetration state from long sequences of welding image signals, adjusting welding power, and stabilizing welding quality.

## 1. Introduction

Laser welding is an indispensable technology in the field of automatic welding [1]. With the increasing popularity of motion mechanisms, large workpieces are gradually processed by laser welding technology, such as aircraft skins, automobile skeletons, and high-speed rail outlines [2]. However, in actual production, a constant welding surface cannot be guaranteed due to welding thermal deformation and manual polishing errors. Consequently, it is challenging to achieve quality monitoring and process control for long-sequence laser welding [3].

**Figure 1:**
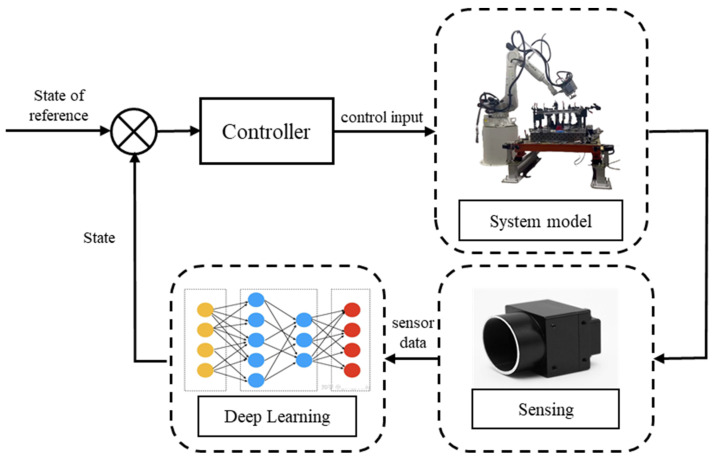
The block diagram of the process control strategy.

Figure 1 depicts a block diagram of the process control strategy based on artificial intelligence. In the control of the laser penetration process, the control strategy and penetration recognition are the keys to ensuring the control effect. Penetration recognition encompasses the selection of sensing equipment and the accuracy and efficiency of the recognition algorithm. Currently, a diverse range of sensing devices is employed for monitoring laser welding penetration [4]. Laser welding penetration based on visual perception is an effective way to improve welding quality [5]. The interaction of the laser with the workpiece surface results in the formation of a molten pool and keyholes. However, due to the inherent characteristics of the laser welding process, obtaining stable and clear images of the molten pool and keyholes poses a significant challenge. Laser monitoring technology based on visual sensing is divided into paraxial visual sensing and coaxial visual sensing according to different installation methods [6]. Coaxial visual surveillance systems are widely used in laser welding tasks due to their ease of installation and active observation of weld pool dynamics [7]. The interference of strong light and plasma generated in the laser welding process makes it difficult to extract stable molten pool and keyhole features. The development of deep learning improves the stability and generalization of feature extraction.

Deep learning performs exceptionally well in the laser welding penetration recognition process, extracting key features through multiple hidden layers to accurately identify the state of the welding process. Numerous studies have demonstrated that deep learning exhibits extraordinary potential in laser welding penetration recognition. However, recognition methods based on deep learning under laser welding process control still suffer from the following limitations:

1. The limited computational resources available at the edge of the control system constrain the efficiency of laser welding image feature extraction processes.
2. The welding process is a time series process, and the acquired images fluctuate continuously in time. Short-term noise is not favorable for laser welding recognition that relies on long-time information and can reduce the accuracy of melt-through recognition.
3. Fusion recognition manifests itself as a cumulative effect of the image signal over time. The cumulative contribution is the process of continuously extracting similar features at critical times and enhancing the contribution to the recognition in the time dimension. RNN (Recurrent Neural Network) results do not handle the problem well.

This paper proposes a laser penetration recognition method that is based on a two-stage temporal convolutional network. Additionally, a simple closed-loop control experiment is designed to verify the feasibility of the proposed method within a process control system. The main contributions of this paper are as follows:

1. This study proposes a lightweight segmentation model based on channel pruning technique to extract molten pool and keyhole features, improving the model’s accuracy and computational efficiency.
2. This study proposes ST-TCN (temporal convolutional network based on attention mechanism) to improve the efficiency and accuracy of long-term information penetration recognition. The parallel model structure was used to improve the scope of model-aware time series. This method provides a new idea for the problem of long-sequence information fusion penetration recognition.
3. We designed welding penetration and closed-loop control experiments involving plates of unequal thickness. The experimental results demonstrate that the proposed penetration identification method exhibits excellent performance in both welding power control and long weld penetration identification.

## 2. Related Work

### 2.1. Feature Extraction Based on Lightweight Models

In order to simulate the welding process in which the welder predicts the weld penetration state from the weld image, researchers have tried using both a one-stage model and a two-stage model [8,9]. The single-stage model takes the weld image as input and outputs the penetration recognition state, thereby enabling end-to-end model training [10,11,12,13]. This method eliminates intermediate subtasks and is conducive to maximizing global optimization. However, the inexplicability of deep learning [14] makes it challenging to pinpoint problems within the model during the recognition process. The existing interpretability methods deviate from the actual model reasoning, and the one-stage model exacerbates this uncertainty. The two-stage model includes feature extraction and classifier design [15,16,17,18]. Traditional image processing algorithms [15,16] or convolutional neural networks [17,18] have been used to extract welding features such as molten pool width, molten pool area, and keyhole frequency. Traditional machine learning techniques, including support vector machines, random forests, and depth perception, have been employed as classifiers [19,20,21,22] to classify welding penetration. In recent years, to reduce model complexity, researchers have achieved model lightweight by constructing compact network structures [23] and incorporating attention mechanisms [24]. However, despite these efforts, compact lightweight models still struggle to meet the real-time performance requirements for edge-end inference.

### 2.2. Penetration Identification Based on Time Series Modeling

The welding process is a dynamic process that changes with time, and researchers add time dimension information to improve the accuracy of molten pool identification. Molten pool recognition based on sequential welding images includes recurrent neural networks based on LSTM and GRU, 3D convolutional neural networks, and image fusion methods. The image fusion method fuses images of different time points and achieves fusion recognition in the time dimension by compressing the time dimension information. Hong et al. [25] and Cheng et al. [26] proposed a feature-level fusion strategy and pixel-level fusion strategy, respectively, to achieve fusion recognition of long-term dynamic information through welding input. This method improves the amount of image information and image quality, but the low computational efficiency cannot meet real-time requirements. The use of a 3D convolution kernel to extract time series features is also a key method for fusing time series information, which suffers from the problem of a large number of parameters and low computational efficiency. For this reason, Yang et al. [27] proposed to use fealty-level 3DCNN to recognize the welding state of high-speed railway aluminum alloy materials, which is more efficient than data-level 3DCNN, and the processing speed of the online monitoring system is 10 HZ. Liu et al. [28] proposed 3DSMDA-Net to reduce the size of the model by improving the network structure of 3DCNN. Researchers proposed a lightweight method to alleviate the problem of low processing efficiency of 3DCNN, but this method cannot realize the problem of welding penetration recognition that depends on information for a long time. Time series models based on LSTM [29,30,31] and GRU [32] are used to solve the problem of time information fusion for melt penetration recognition. Although the above methods have made progress in the field of laser welding monitoring based on time series, they cannot rely on the operation efficiency of long-term information.

## 3. Methodology and Analysis

### 3.1. Experimental Platform and Materials

**Figure 2:**
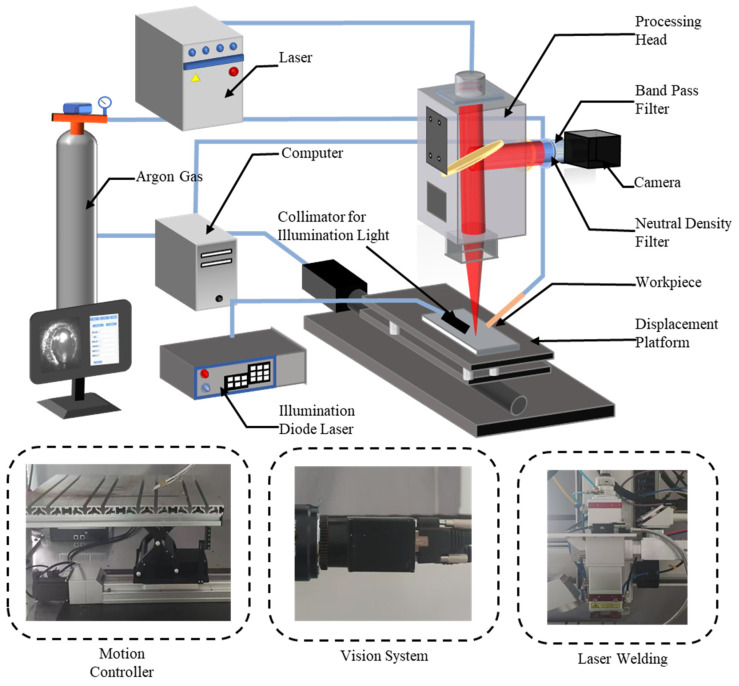
Diagram of the laser welding monitoring system.

The laser welding experimental system mainly includes a laser welding head, a uniaxial moving platform, a shielding gas (argon) device, and an image acquisition system, as shown in Figure 2. The MFCS-4000W laser system is connected to the LD150 4KW (Shanghai Gaize Laser Technology Co., Ltd., Shanghai, China) laser welding head by optical fiber. A single-axis motion platform was employed for mobility during the experiment. Argon can effectively suppress plasma interference. A camera is mounted on the processing head and coaxially aligned with the laser beam through a partially transmissive mirror. An exposure time of 38 microseconds is selected to improve the molten pool and keyhole detail. Coaxial monitoring faced significant challenges due to interference from illumination and fluctuation in the molten pool during welding. To address this, integrating a neutral density filter and an 808 nm band-pass filter into the camera lens enabled the capturing of high-quality grayscale images without distortion. A 30 W illumination diode laser, operating at an 808 nm wavelength, illuminated metal oxidation and reduced interference from metal plasma during laser welding, ensuring clear identification of the molten pool and keyhole in system-acquired images.

The experimental materials were HC420 steel plates of 140 mm × 50 mm with thicknesses of 1.0 mm, 1.2 mm, 1.5 mm, 1.8 mm, and 2.0 mm. The protective gas flow rate was 15 L/min, and the visual sensor frequency was 200 FPS. Table 1 presents the main process parameters. Among these, laser power, welding speed, and shielding gas flow rate emerge as the primary variables influencing the melting behavior of the selected plate thicknesses. Conversely, the extent of defocusing and the laser spot size are identified as having secondary effects on the melting outcomes for the given plate thicknesses. After conducting numerous experiments to determine the experimental parameters listed in Table 1, it was observed that 1 mm and 1.2 mm plate thicknesses exhibited varying degrees of non-penetration, 1.5 mm plate thicknesses showed normal penetration, and 1.8 mm and 2 mm plate thicknesses presented burn-through states. Sufficient data were collected using 50 plates with the same process parameters to ensure a diverse dataset and improve the generalization of the system.

### 3.2. Feature Extraction

#### 3.2.1. Images of Laser Molten Pool and Keyhole

**Figure 3:**
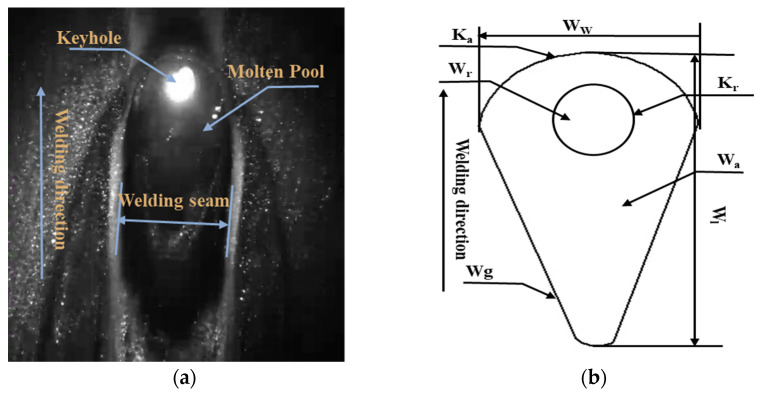
Acquisition of image information: (**a**) image acquisition by visual sensors; (**b**) characteristic parameter definitions.

Figure 3a shows a laser welding molten pool keyhole image acquired by visual sensing at a certain point in time. The image consists of the molten pool, the keyholes, the plasma, and the weld seam. To quantitatively analyze the morphological feature parameters of the molten pool keyholes, the following are shown in the figure: width of the molten pool (WW), length of the molten pool (Wl), area of the molten pool (Wa), angle of the tail of the molten pool (Wg), diameter of the head of the molten pool (Wr), area of the keyholes (Ka), and diameter of the keyholes (Kr). Figure 3b illustrates the detailed specifics of the aforementioned parameters. The core of feature extraction lies in the stable acquisition of the molten pool contour, and the arc light and plasma of the welding process increase the uncertainty of the molten pool segmentation. Deep learning exhibits excellent feature representation capabilities and is able to overcome the lack of robustness demonstrated by traditional algorithms in dealing with noise and complex disturbances in the laser welding process.

#### 3.2.2. Lightweight Image Segmentation Network

**Figure 4:**
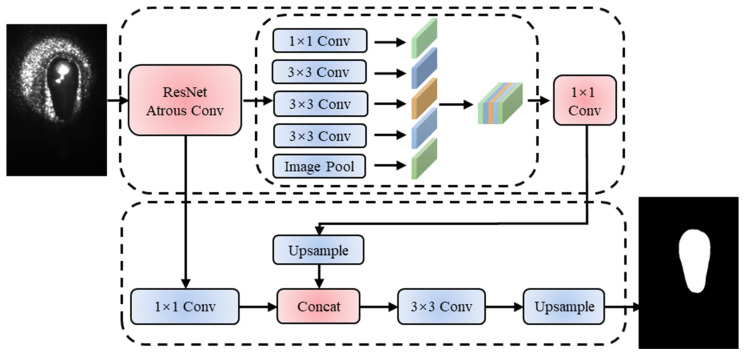
Schematic diagram of DeeplabV3+ model structure.

The DeepLabV3+ model utilizes an encoder–decoder structure, which is commonly employed in segmentation models. In this structure, the encoder adopts a specific architecture as the backbone network to accelerate the computation process. As depicted in Figure 4, the backbone network generates two outputs: high-level features and low-level features. The selection of the backbone network can significantly impact both the segmentation accuracy and the processing efficiency of the model. ResNet introduces a residual structure, effectively addressing the issue of gradient vanishing while training very deep neural networks, thereby enabling better performance. The network architecture of ResNet is straightforward, making it easy to train and implement. In this study, ResNet is employed as the backbone network model to achieve the segmentation effect of DeepLabV3+.

Although the attention transfer-based DeepLabV3+ model can stably segment the molten pool region, the network possesses a large number of parameters and is computationally intensive. To facilitate its application in closed-loop control systems, it is essential to reduce the model’s complexity and parameters. Model lightweight encompasses various methods, including knowledge distillation, pruning, quantization, and attention transfer. The structural design of the model and the choice of the number of layers often lead to redundancy, and model pruning achieves lightweight by identifying and eliminating redundant parameters based on certain criteria.

Pruning techniques are categorized into structural and non-structural pruning. Structural pruning is usually performed with the filter or the entire network layer as the basic unit. Among these techniques, channel pruning [33] reduces the model’s complexity by eliminating unimportant channels and their associated input/output relationships.

**Figure 5:**
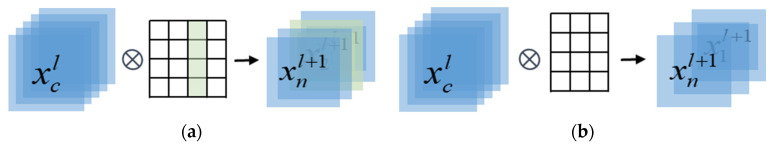
Pruning process of DeeplabV3+: (**a**) model before pruning; (**b**) pruned model.

The channel pruning process is illustrated in Figure 5. Figure 5a depicts the model structure before pruning. In the feature map of the convolution layer of the original network, each channel is associated with a sparse scaling factor. If the scaling factor corresponding to a featured channel is less than a certain threshold, it indicates that the channel’s contribution to the overall performance of the network is minimal. Consequently, the convolution kernel and the associated feature channel in the preceding layer are pruned to achieve model compression. Figure 5b shows the structure of the model after pruning. The scaling factor of the batch normalization (BN) layer is utilized to evaluate the importance of each channel. The formula for the BN layer is expressed as follows:

(1) $$y=\frac{x-E\left[x\right]}{\sqrt{Var\left[x\right]+\varepsilon }}*\gamma +\beta$$

where *x* and *y* are the inputs and outputs; *γ* and *β* are the scale factor and bias factor, respectively. *E*(*x*) represents the expectation of the input data, and *Var*[*x*] is the variance of the input data.

The loss function is as follows:

(2) $$L=l\left(w,b\right)+\lambda \sum \left|\gamma \right|$$

where *w* and *b* are network parameters, *l*(.) is the original loss function, and *λ* is the weight of the *L* regularization term.

The specific steps of channel pruning in this study consisted of the following major steps:

1. Modify the network structure of DeeplabV3+, the inputs and outputs of the channels of ResNet and the layers connected to the backbone network are fixed, and the fixed channels are modified with variable channel variables.
2. Sparse training, with *L* regularization constraints imposed on the channel layers in the model, promotes model sparsification and separates unimportant channels.
3. Pruning: After completing sparse training, set the pruning rate and delete the corresponding channel information when the corresponding channel weight is less than the set pruning rate. Generate a parsimonious model that occupies less space.
4. Fine-tuning. The accuracy of the pruned model is too low and needs to be retrained to obtain new weight parameters.

**Figure 6:**
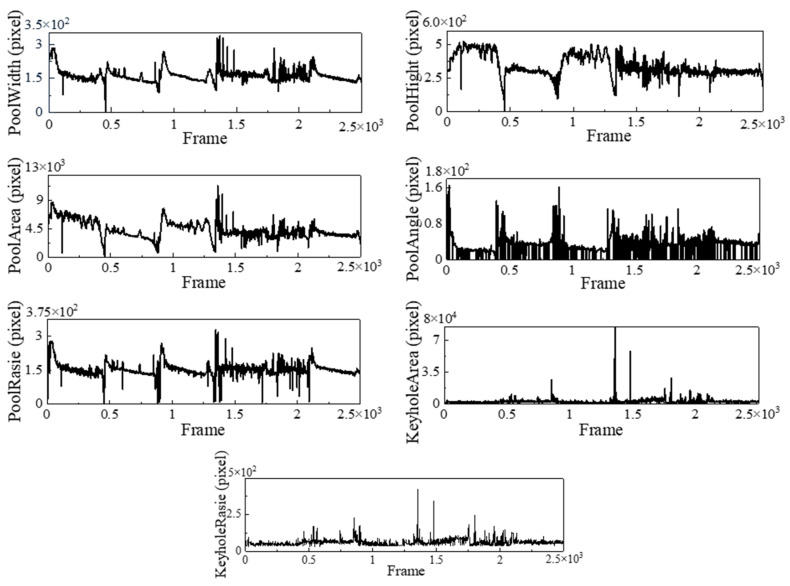
Characteristic curves of molten pool and keyholes.

The molten pool and keyhole images are segmented to obtain accurate molten pool and keyhole contours. In this paper, traditional geometric image processing is selected to obtain seven feature parameters. Figure 6 shows the feature extraction results after the segmentation model processing. The images for feature extraction are selected from the test set with three fused states, and the data obtained from segmentation are subjected to geometric knowledge to extract the key features.

### 3.3. Penetration State Recognition Based on ST-TCNs

#### 3.3.1. TCN Model

**Figure 7:**
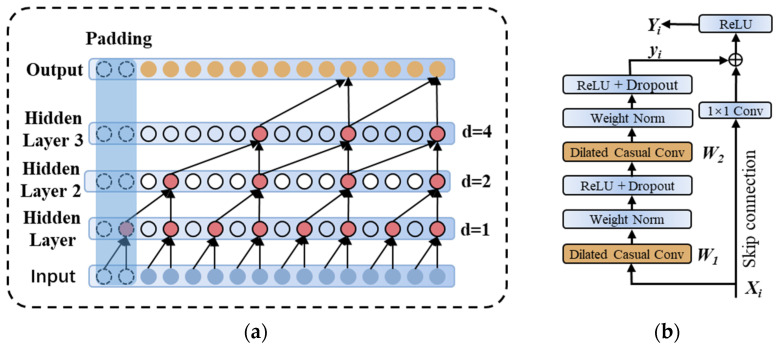
TCN architecture: (**a**) dilated casual conv; (**b**) TCN block.

The TCN model, first proposed by Bai [34], is a time series data processing model based on CNNs that incorporates improved optimization—causal convolution, dilated convolution, and residual connectivity. Compared to other theoretical models, TCN sets up a strict historical causality in the training of time series data, effectively addressing the problem of existing models struggling with weak time series classification for molten pool and keyhole time series data. The dilation factor ensures that the size of the hidden layer remains the same as the input size through the use of zero padding, and the output at the current time step is derived from the progression of historical information. As shown in Figure 7a, the output of the hidden layer’s convolutional operation is used as the input for the next convolutional layer, building upon the output of the previous convolutional layer.

Cavity convolution calculates the size of the specified steps skipped at each layer through the cavity expansion factor, which in turn results in larger input information at larger filter sizes. When performing a convolutional computation, the model exponentially increases the dilation factor with depth. The outputs can all be traced back to the input in a similar way to the T-moment data, indicating that this arrangement ensures that the convolution kernel efficiently computes all layers and specific inputs in the history relation. The network is able to feel the exponential growth of each layer and efficiently utilize the input of historical data.

Figure 7b shows the block in TCN. TCN further improves the classification accuracy of the temporal task by increasing the network depth. The use of residual networks instead of layer-to-layer connections can speed up the training process, which is good for solving the problem of disappearing gradients in deep networks and maintaining long-history information.

#### 3.3.2. Attention Mechanisms

**Figure 8:**
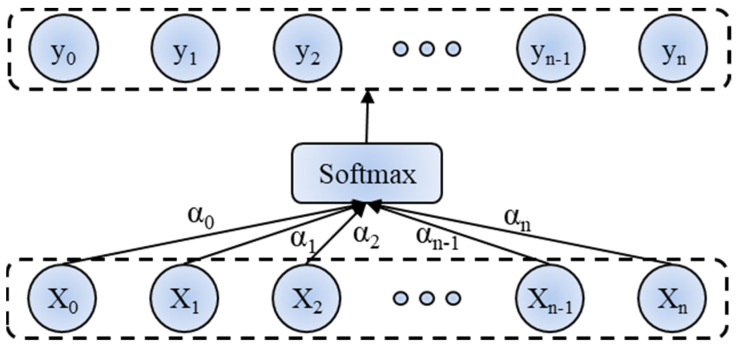
Structure of the attention mechanism.

The attention mechanism calculates the similarities between the element and other elements and normalizes these similarities into attention weights. The structure of the attention mechanism is shown in Figure 8. The feature signals are somewhat noisy and partially redundant. To adequately extract representative local features, the temporal attention mechanism focuses on the weight distribution of different time steps in the sequence, and the spatial attention mechanism performs a weighted sum of different feature sequences to strengthen the spatial correlation. The calculation process is as follows:

First, the $a_{n}$ generation of attentional weights is as below:

(3) $$a_{n}=W_{c}x_{n}+b_{c}$$

where *W_c_* and *b_c_* are the weights and biases, respectively. Next, $y_{n}$ is obtained by normalizing $a_{n}$:

(4) $$y_{n}=\frac{\exp(a_{n})}{\sum_{i=1}^{n+1}\exp(a_{n}^{\left(i\right)})}$$

#### 3.3.3. ST-TCNs

**Figure 9:**
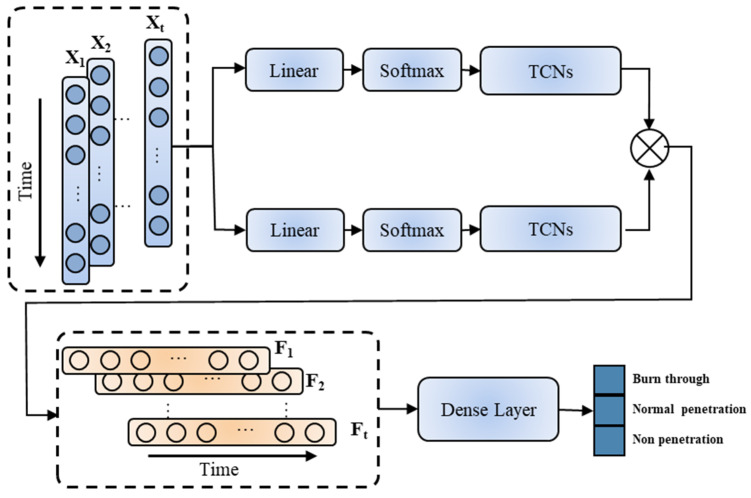
ST-TCN model architecture diagram.

The architecture of the ST-TCN is shown in Figure 9, which consists of a spatial and temporal attention module integrated with temporal convolutional networks. The changes in the molten pool and keyhole features are reflected in the time dimension and feature dimension. Therefore, the model extracts learned features using the spatial and temporal attention mechanisms, and these extracted feature data are then input into the TCN model to fuse historical information, thereby achieving the construction of model features. The ReLU unit increases the sparsity of the network and helps moderate overfitting. Dropout discards neurons from the network according to a certain proportion to reduce the risk of overfitting. Additionally, the residual model structure reduces the risk of overfitting between layers. Finally, the two features are fused and classified by a classifier.

## 4. Results

### 4.1. Training and Validation of Lightweight Segmentation Models

Model training is based on the Pytorch framework, and the Ubuntu18.04 system was selected for the training and testing platform. Nvidia GeForce GTX2080 GPUs were selected for model training, and Nvidia Jetson TX2 was selected for inference. Segmentation accuracy evaluation metrics were selected as dice, IoU, MAE, recall, precision, and F1. The inference speed of the model was measured using FLOPs and FPS. The dataset was obtained after the processing scheme in Section 3.1, with a total of 43,500 samples, including 30,450 training sets and 3050 test sets.

**Figure 10:**
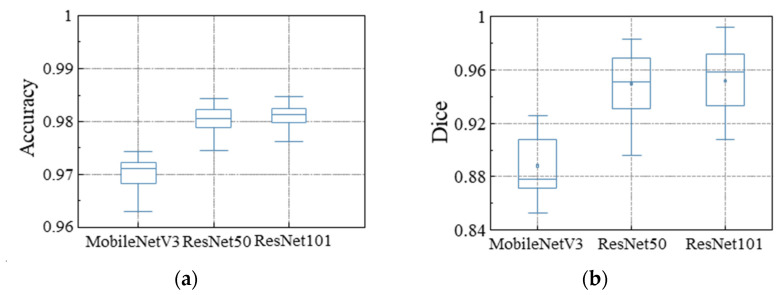
Training result of different backbone network models: (**a**) accuracy of segmentation; (**b**) dice of segmentation.

MobileNetV3, ResNet50, and ResNet101 are selected as backbone networks for training in this study. The number of training rounds is selected as 200, the momentum value is selected as 0.9, and the weight decay is 0.0001. Figure 10a shows the accuracy of different backbone network models. This is related to the lightweight model structure, which causes the model to lack generalization performance for some disturbed images. Figure 10b presents a comparison of the dice coefficients achieved by various backbone network models. As the number of layers increases, the accuracy and dice values of the ResNet series increase, and the model exhibits higher segmentation accuracy, smaller variance, and higher robustness. However, the increase in parameters can affect the inference efficiency of the model. Therefore, ResNet50 is more suitable as the backbone network for this study.

**Figure 11:**
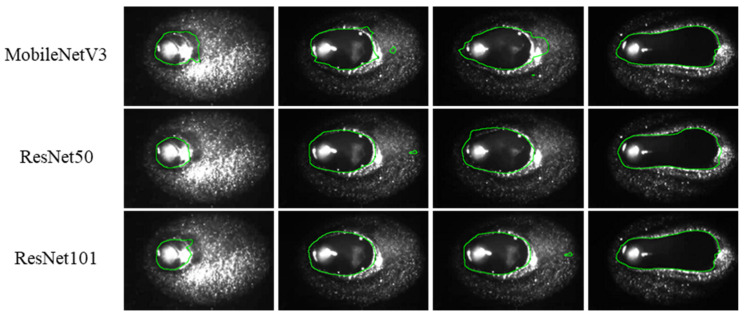
Detailed diagram of molten pool segmentation for different backbone networks.

Figure 11 shows the segmentation results of different backbone networks. The segmented contours of MobileNetV3 deviate from the real molten pool contour, which affects the accuracy of feature extraction. The segmentation results of ResNet50 and ResNet101 do not have significant deviations from the real molten pool contour, whereas the segmentation results of ResNet101 exhibit overfitting in the initial state of welding. In this study, ResNet50 is chosen as the backbone model for subsequent analysis and validation.

**Figure 12:**
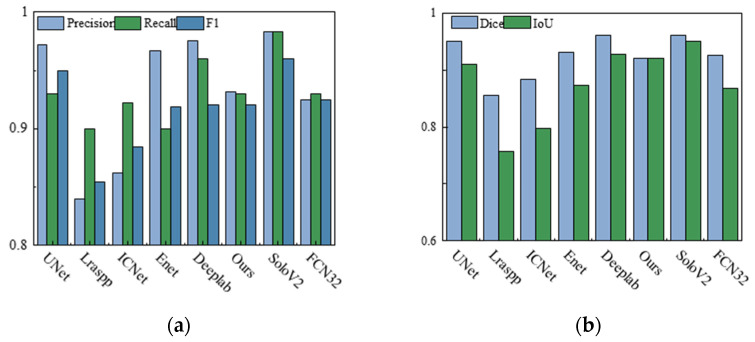
Evaluation of different segmentation methods: (**a**) precision, recall, and F1 of models; (**b**) dice and IoU of models.

The currently stable segmentation models were selected to test the segmentation of the molten pool. The lightweight segmentation models include UNet, Lraspp, ENet, FCN, ICNet, SoloV2, and DeeplabV3+. Figure 12a shows the precision, recall, and F1 scores of the training results for the different models. The accuracy of Lraspp, ENet, FCN, and ICNet is lower compared to UNet and SoloV2, while the pruned network has similar accuracy to UNet and SoloV2. Figure 12b illustrates the performance of various models in terms of their dice similarity coefficients and intersection over union based on the training outcomes. The segmentation accuracy of these models is slightly lower than that of UNet and SoloV2, but the number of model parameters is also lower.

We further tested the image processing speed of the two networks on the edge device, and the results are shown in Table 2. The inference speed at the edge-end is 2.15 times faster than that of the original model, reaching a processing speed of 49 frames. The experimental results show that the method in this paper meets the premise of the accuracy of segmentation and achieves faster processing speed at the edge-end to meet the real-time requirements of feature extraction.

### 4.2. Training and Validation of ST-TCNs

The molten pool and keyhole features extracted from more than 50 welds as described above were selected to be divided into different time signals with different time windows as the dataset and test set. The dataset with the name welding feature.ts was uploaded to the supplementary material. Among them, 70% were selected as the training set and 30% were selected as the test set. The labels are 0, 1, and 2 for unfused, normally fused, and over-fused, respectively.

**Figure 13:**
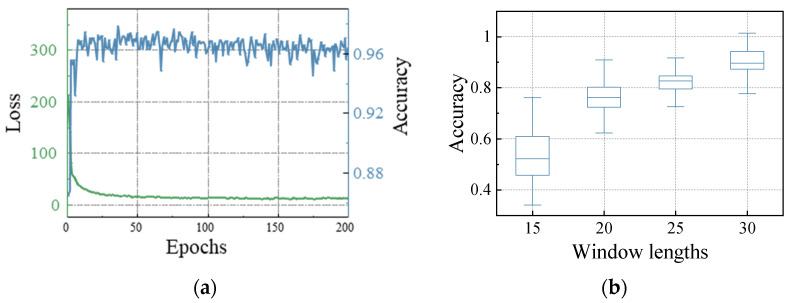
ST-TCN test results. (**a**) Training process. (**b**) Accuracy of different lengths.

The experimental equipment listed in Section 3.1 was chosen for the experiment. Cross-entropy was chosen as the loss function for training, and accuracy was used to measure the effectiveness of the model classification. Figure 13a depicts the training progression of the ST-TCNs.

The window lengths of 15, 20, 25, and 30 were chosen as the time windows. Figure 13b shows that a window length of 25 performs better in terms of segmentation accuracy and variance distribution, and the loss function converges quickly with less accuracy oscillation.

**Figure 14:**
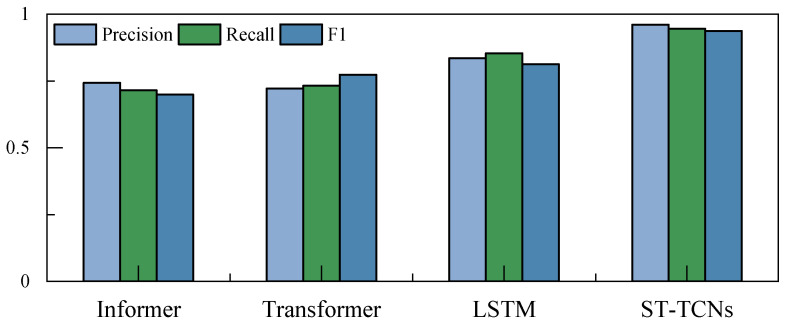
Classification accuracy of the proposed method and other methods.

The comparison experiments were also conducted with a window size of 25, and precision, recall, and F1 were used as measures of classification accuracy. Figure 14 shows that with the same time window, the ST-TCNs have the highest classification accuracy, and the overall performance is superior. The LSTM network structure itself is not suitable for the long time series classification problem, and a transformer-type network structure is not applicable to the problem in this study.

### 4.3. Long-Time Sequence Welding Penetration Recognition Experiment

**Figure 15:**
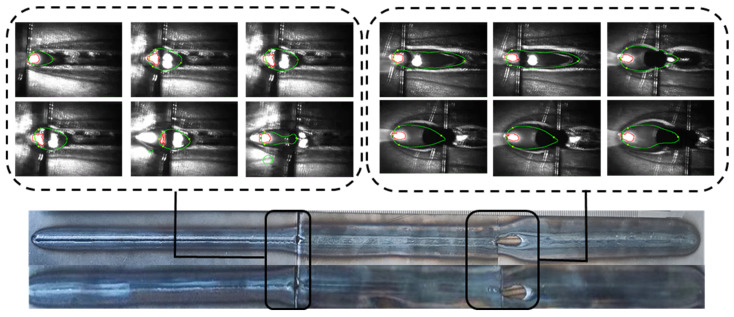
HC420 welds in different penetration states.

To verify both the versatility of the method presented in this paper and its recognition accuracy in actual processing scenarios, it is necessary to conduct laser welding experiments for head and tail splicing using workpiece materials of varying thicknesses. The workpiece materials used had thicknesses of 1 mm, 1.5 mm, and 2 mm. The welding speed was set to 13 mm/s, the welding power was set to 2400 W, and the physical appearance of the weld seam is illustrated in Figure 15. The black areas signify the transitional phases of the welding process, where changes in thickness result in alterations to the welding state due to energy accumulation.

**Figure 16:**
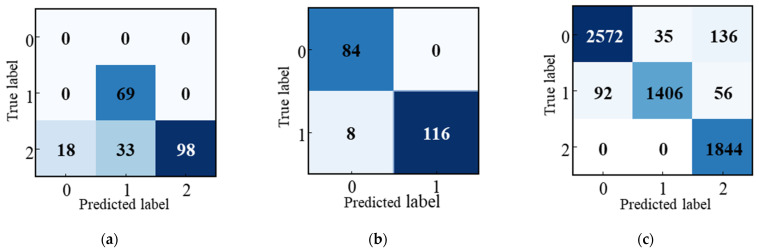
Confusion matrix: (**a**) burn-through to the normal penetration stage; (**b**) normal penetration state to non-penetration stage; (**c**) the whole weld stage.

A total of 6187 frames were acquired in the experiment, of which 2746 had non-penetration status, 1554 had normal penetration status, and 1887 had burn-through status. Figure 16 depicts the confusion matrix for the different stages. Figure 16a illustrates the confusion matrix parameters for the transition from the burn-through stage to the normal penetration stage, achieving a correct identification rate of 76%. Figure 16b shows the confusion matrix parameters for the transition from the normal penetration state to the non-penetration state, with a correct identification rate of 96.1%. Lastly, Figure 16c presents the confusion matrix for the entire weld stage, achieving an overall correct identification rate of 98.96%.

**Figure 17:**
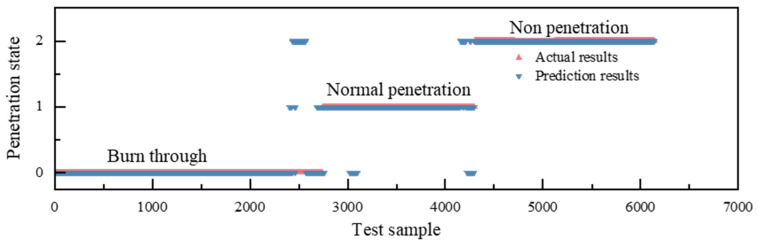
Difference between recognition results and actual results of each penetration state.

Figure 17 shows a plot comparing the weld state recognition with the real weld state. In the stable stage of welding, the accuracy of weld penetration state recognition is relatively high, whereas the recognition rate is poor in the transition stage. The training set is captured from images of the steady-state molten pool, and the melt penetration classification recognition model is also based on steady-state recognition. In the transition phase, the keyhole is segmented between two plates, resulting in keyhole parameters that deviate from the standard keyhole size. Additionally, the shape of the molten pool caused by the keyholes leads to errors in segmentation accuracy.

### 4.4. Laser Weld Process Control Experiment

**Figure 18:**
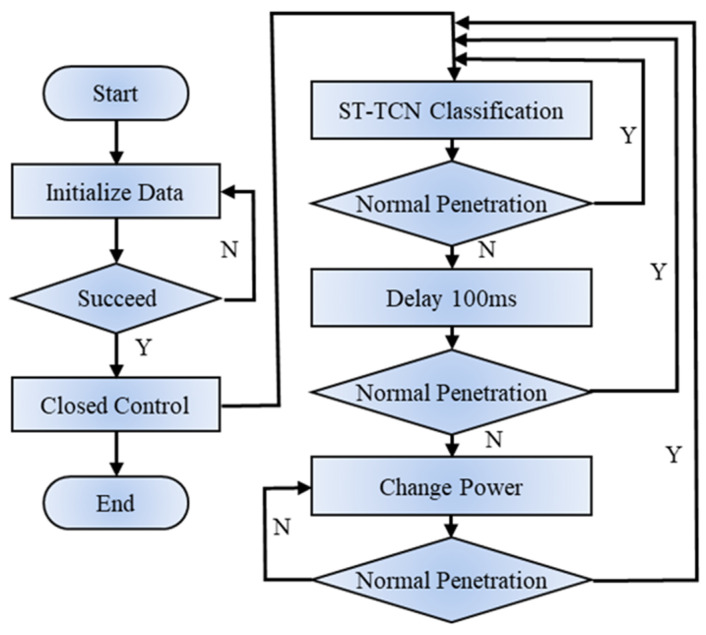
Laser welding power control flow.

The control flow is illustrated in Figure 18, where the model is pre-trained and achieves an inference speed of 20.4 ms. The welding power serves as the control variable, and to simplify the control system design process, the welding process is neither physically modeled nor systematically identified. When the welding state transitions from a normal welding state to a non-normal welding state, adjustments are made to the welding power. Specifically, if the welding state is monitored as a fusion state, the welding power is reduced in increments of 200 W. Conversely, if the welding status indicates non-fusion, the welding power is increased in increments of 200 W. A threshold of five consecutive welding states with the same classification is used to trigger a change in welding power, although there may be some inaccuracy due to the welding state monitoring. If the same welding state occurs three times in a row and differs from the previous stage, the welding power is adjusted accordingly. It is ensured that the original welding parameters are maintained if the condition is met slightly differently.

The experiments were carried out on plates of unequal thickness, and the open-loop and closed-loop experimental processes were designed separately. The initial welding power was set to 2400 W, the welding speed was 15 mm/s, and the protective gas flow rate was set to 15 L/min. The thicknesses of the workpiece materials were 1.5 mm and 2 mm.

**Figure 19:**
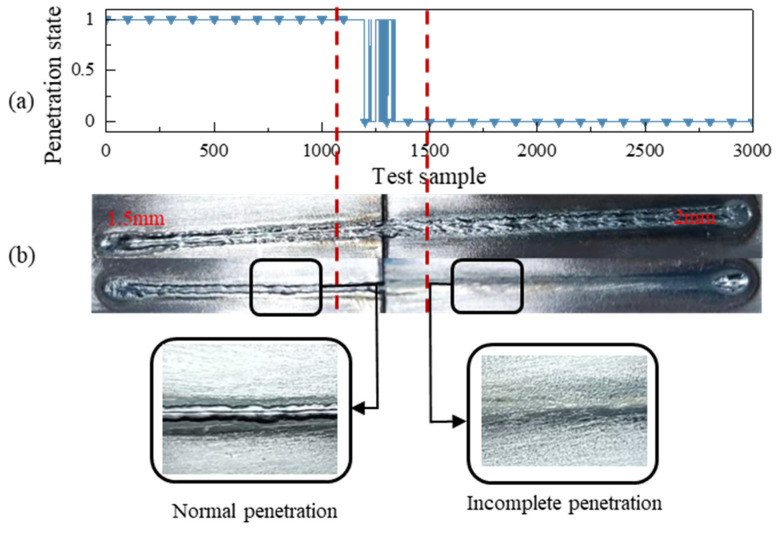
Laser welding penetration experiment: without control: (**a**) change in penetration; (**b**) surface of welding.

Figure 19 illustrates the effect of laser welding without the control process. Figure 19a displays the recognition results obtained by the laser welding penetration recognition algorithm for plates of unequal thickness. During the experiment, the welding parameters remained constant while the plate thickness transitioned from 1.5 mm to 2 mm. Consequently, the weld condition shifted from normal penetration to non-penetration due to the consistent input welding energy. Figure 19b showcases the fused surface quality of the unequal-thickness plates. The experiments revealed the presence of non-penetration defects in the 2 mm plates.

**Figure 20:**
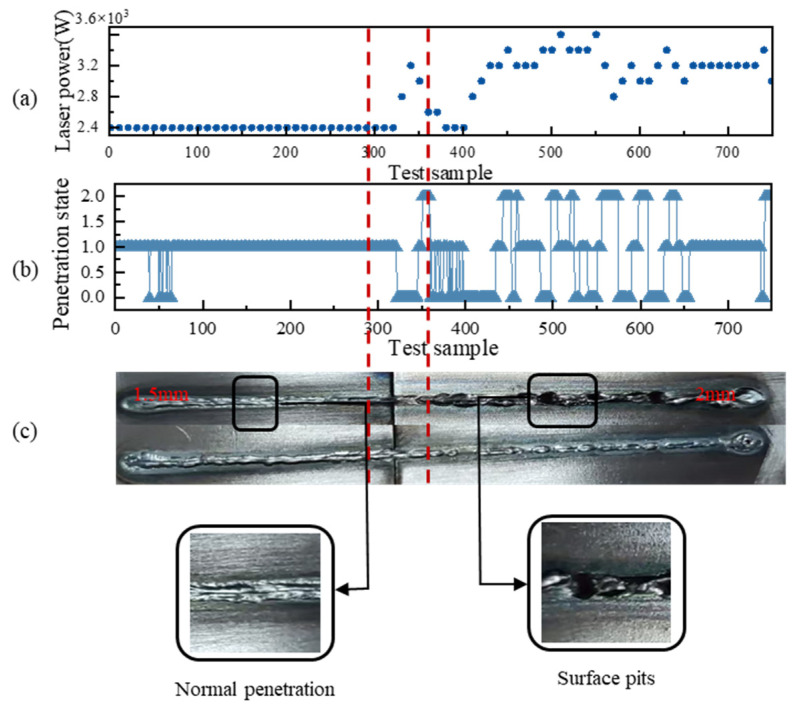
Laser process control welding experiment based on LSTM: (**a**) change in power; (**b**) change in penetration; (**c**) surface of welding.

Figure 20 presents the results of the welding control experiment based on LSTM. Figure 20a demonstrates the power variation during the welding process. When the laser passes through the 2 mm experimental plate, the laser power appears to be regulated erratically, with the welding power fluctuating between 2800 W and 3600 W. Figure 20b displays the recognition results of the welding process recognition algorithm. The recognition process fusion recognition state varies between three fusion states. Figure 20c shows the processing results of the actual weldment. When welding the 1.5 mm welded plate, the weld state is relatively stable, and no obvious defects appear. In contrast, crater defects appeared at the 2 mm experimental plate stage. The rapid fluctuation of welding power led to unstable changes in the molten pool and keyholes, which subsequently resulted in defects.

**Figure 21:**
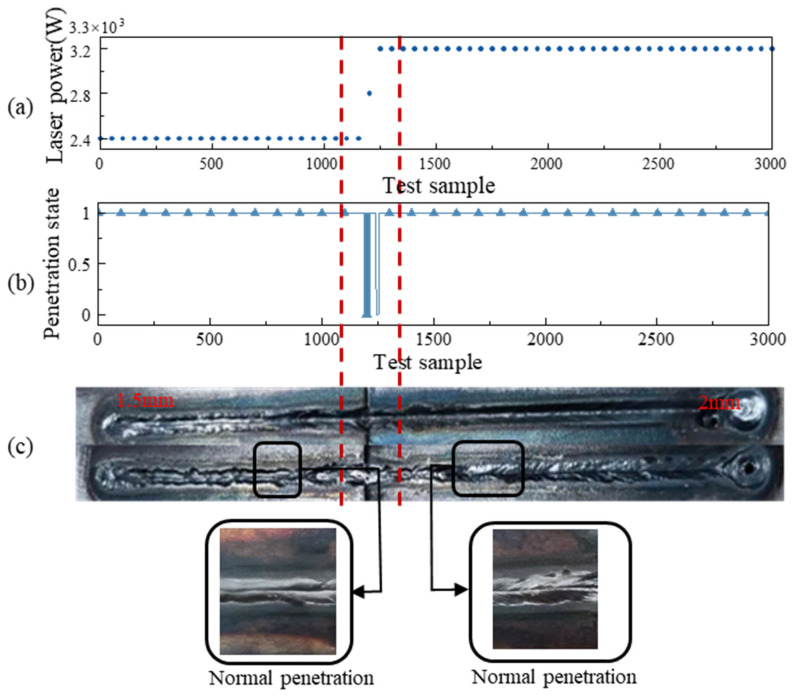
Laser process control based on two-stage temporal convolutional network: (**a**) change in power; (**b**) change in penetration; (**c**) surface of welding.

Figure 21 displays the results of laser process control based on a two-stage temporal convolutional network. Figure 21a illustrates the power variation during the welding process. The initial setting of the welding power is 2400 W. After monitoring the welding state, which transitions from a fused state to an unfused state, the welding power increases in steps. After 0.5 s, when the welding power reaches 3200 W, the welding state is restored to the fused state. Figure 21b presents the recognition results obtained by the recognition algorithm during the welding process. Figure 21c shows the surface weld quality after completion. It can be observed that the entire weld achieves normal penetration without any obvious defects.

## 5. Discussion

In this study, a lightweight image segmentation method based on channel pruning is proposed to achieve stable and efficient extraction of molten pool and keyhole features. Choosing the appropriate base network can lead to better model accuracy and complexity performance. Therefore, studying the segmentation effect under different networks (see Figure 10, Figure 11 and Figure 12 and Table 2) effectively balances segmentation accuracy and efficiency. In addition, the method removes redundant model parameters from the classical segmentation network through the channel pruning technique, which facilitates model deployment at the edge and saves computational resources there.

Then, the ST-TCN model is proposed and applied to long-time-series laser welding penetration classification. This method overcomes the limitation of existing penetration recognition methods, which struggle to handle long-term accumulated information when dealing with large structural components, by selecting the TCN network as the base network [26]. In addition, by introducing the attention mechanism, the method can focus more on the features that require greater attention, thereby enabling more efficient recognition of key acquisition features. In this study, different window sizes are set to explore the impact of data sources on the model’s performance, ensuring that the model can achieve better results in laser welding penetration recognition.

On the other hand, the method presented in this paper achieves very good recognition results for real laser welding scenarios. A very high recognition rate is achieved during the stable welding phase, whereas some recognition deviation occurs in the transition phase due to the deformation of the molten pool and insufficient data sources. This finding is of great significance for the continuous development of laser welding of large structural parts. Furthermore, laser control experiments have demonstrated that the method can achieve stable and high-precision recognition of penetration results, providing a solid basis for improved process control in laser welding. However, for continuous high-frequency melt-through recognition scenarios, the rapidity of the control strategy proposed in this paper may not be guaranteed, necessitating further research.

## 6. Conclusions

This paper investigates a two-stage time series network-based method for weld penetration state identification in a laser welding process control system. A lightweight segmentation model, based on channel pruning, is proposed to achieve edge-extractable features of the molten pool and keyhole. Furthermore, ST-TCNs is introduced to realize weld penetration identification in long-time-series laser welding processes. The main conclusions of this study include the following:

1. We built a coaxial laser sensing monitoring system to obtain clear images of the molten pool keyholes and designed different welding schemes to obtain datasets under different welding conditions.
2. A lightweight segmentation model was proposed, based on the channel pruning technique, to achieve real-time and stable segmentation of molten pool and keyhole shapes, as well as feature extraction for edge devices. The model achieves a segmentation accuracy of 95.96% and enables edge-side inference at 49 FPS.
3. A temporal convolutional network incorporating time-space attention was introduced to classify melting states in a long-term laser welding process. The optimal time window for this purpose was determined through experimentation.
4. Using the unequal thickness plate as the experimental object, we designed both a laser weld penetration identification experiment and a process control experiment. The proposed model robustly identifies the through state with an accuracy of 98.96% and an inference time of 20.4 ms. In the process control experiment, the adjustment time was 0.5 s, allowing for consistent welding molding.

While some progress was made in penetration identification for laser welding process control based on the current results, several aspects remain to be explored in the future: Firstly, more applicable control strategies are needed to facilitate rapid adjustment of laser welding parameters, ensuring more stable control outcomes. Secondly, increasing the number of sensors would enhance the generalization of the monitoring process and broaden the range of defects that can be detected. Lastly, the stability of the recognition algorithm must be further investigated to accommodate complex deformations on the structure’s surface.

## Figures and Tables

**Table 1:** Welding experiment parameters.

Welding Parameters	Value
Welding speed	15 mm/s
Laser power	2400 W
Gas flow rate	15 L/min

**Table 2:** Model complexity and inference speed before and after model pruning.

Model	Flops (G)	Params (M)
Ours	10.8	11.5
DeeplabV3+	44.28	46.80

## Data Availability

The authors confirm that the data supporting the findings of this study are available within the supplementary materials.

## Supplementary Materials

The following supporting information can be downloaded at: https://www.mdpi.com/article/10.3390/ma17184441/s1, Supplementary File: welding feature.ts: the molten pool and keyhole features extracted from more than 50 welds.
